# Hyperspectral Analysis of Leaf Pigments and Nutritional Elements in Tallgrass Prairie Vegetation

**DOI:** 10.3389/fpls.2019.00142

**Published:** 2019-02-25

**Authors:** Bohua Ling, Douglas G. Goodin, Edward J. Raynor, Anthony Joern

**Affiliations:** ^1^School of Civil and Transportation Engineering, Guangdong University of Technology, Guangzhou, China; ^2^Department of Geography, Kansas State University, Manhattan, KS, United States; ^3^Agricultural Research Service, Rangeland Resources & Systems Research Unit, Fort Collins, CO, United States; ^4^Division of Biology, Kansas State University, Manhattan, KS, United States

**Keywords:** remote sensing, hyperspectral analysis, leaf pigments, nutritional elements, tallgrass prairie

## Abstract

Understanding the spatial distribution of forage quality is important to address critical research questions in grassland science. Due to its efficiency and accuracy, there has been a widespread interest in mapping the canopy vegetation characteristics using remote sensing methods. In this study, foliar chlorophylls, carotenoids, and nutritional elements across multiple tallgrass prairie functional groups were quantified at the leaf level using hyperspectral analysis in the region of 470–800 nm, which was expected to be a precursor to further remote sensing of canopy vegetation quality. A method of spectral standardization was developed using a form of the normalized difference, which proved feasible to reduce the interference from background effects in the leaf reflectance measurements. Chlorophylls and carotenoids were retrieved through inverting the physical model PROSPECT 5. The foliar nutritional elements were modeled empirically. Partial least squares regression was used to build the linkages between the high-dimensional spectral predictor variables and the foliar biochemical contents. Results showed that the retrieval of leaf biochemistry through hyperspectral analysis can be accurate and robust across different tallgrass prairie functional groups. In addition, correlations were found between the leaf pigments and nutritional elements. Results provided insight into the use of pigment-related vegetation indices as the proxy of plant nutrition quality.

## Introduction

Interactive processes among fire, macro grazers, and vegetation canopy are of particular interest in grassland science (Anderson, 2006; Anderson et al., 2007; Allred et al., 2011a,b; Joern and Raynor, 2018). To address critical research questions concerning the scale-dependent, hierarchical processes inherent to grassland systems, it is essential to understand the spatial distribution of canopy characteristics over an extensive area (Wallace et al., 1995; Collins and Smith, 2006; Bartlam-Brooks et al., 2013). With the development of multiple airborne and satellite sensors, there is now a widespread interest in mapping canopy characteristics through remote sensing analysis (Mutanga et al., 2004a; Kawamura et al., 2008; Trombetti et al., 2008; Ozyigit and Bilgen, 2013). Compared to traditional manual field measurements, remote sensing provides a way to rapidly and cost-effectively collect canopy information such as nutritional status, photosynthesis rates and canopy structure over a large vegetative area (Asrar et al., 1992; Chen and Cihlar, 1996; Gitelson et al., 1996; Coops et al., 2003; Belluco et al., 2006). Of course, retrieving such canopy characteristics from remotely sensed data requires analytical methods capable of converting spectral response data into usable information.

Spectral analysis at the leaf level is a preliminary step to extending remote sensing of vegetation characteristics at the canopy level. The leaf-level spectral analysis provides a fast and cost-effective method of detecting foliar pigments and nutritional elements (Carter and Knapp, 2001; Mutanga et al., 2004a; Blackburn, 2007). In plant hyper-spectroscopy, the visible and near infrared spectral region (400–2500 nm) is of special interest. Hyperspectral analysis in this region is often based on the spectral features resulting from absorption of electromagnetic energy by a variety of chemical bonds in the leaf organic matter. The foliar pigments and nutritional elements can be estimated from the spectral features due to their direct or indirect associations with the leaf organic matter (Goetz et al., 1985; Clark et al., 2003; Galvez-Sola et al., 2015).

Vegetation characteristics can be linked to spectral features statistically. In hyperspectral remote sensing, spectral data are typically high-dimensional, fine spectral bands which are highly correlated with each other (Landgrebe, 2002). High correlations among a large number of predictor variables (hyperspectral bands) may lead to problems of multicollinearity and overfitting when using conventional multivariate regression for empirical modeling (Kumar, 1975; Hawkins, 2004). In contrast, partial least squares (PLS) regression addresses multicollinearity and overfitting properly, and is therefore widely used in hyperspectral analysis (Li et al., 2014; Yu et al., 2015; Ryan and Ali, 2016). PLS regression can be considered a supervised dimension reduction technique, which takes into account correlations between the predictor variables and the dependent variables. Through PLS regression the predictor variables are transformed into latent factors in directions associated with the maximum variance in the dependent variables (Malthouse et al., 1997; Rosipal and Trejo, 2002). Usually, the first few latent factors explain most of the variance in the dependent variables, and thus the dependent variables can be modeled by a reduced number of latent factors. In a PLS regression model, the model explanatory power increases as the number of PLS factors increases. However, the model prediction accuracy may decrease with an increase in model complexity (Kuhn and Johnson, 2013).

As an alternative to empirical methods, vegetation characteristics can also be retrieved through inverting physical models of plant radiative transfer (Goel and Thompson, 1984a,b; Goel and Grier, 1988). Compared to empirical methods, physical models provide a more systematic description of relationships between vegetation characteristics and vegetation reflectance, which are potentially more robust and universal across different measurement conditions, vegetation types and study sites. In remote sensing of vegetation, PROSPECT is one of the most popular leaf-level models due to its ease of use and general robustness. In the PROSPECT model, leaf reflectance and transmittance are modeled simply with the leaf mesophyll structure and biochemical contents (Jacquemoud and Baret, 1990). The leaf biochemical constitutes include chlorophylls, water and dry matter. More recently, carotenoids have been separated from chlorophylls in the latest version PROSPECT 5, which allows more accurate estimations of plant photosynthetic pigments (Feret et al., 2008).

The objective of our study is to estimate leaf pigments and macronutrients across different plant functional groups (grasses *vs*. forbs) in a tallgrass prairie using hyperspectral reflectance data, which is part of a larger research project aimed at understanding the interplay between grassland forage quality and pyric herbivory in a tallgrass prairie. The leaf pigments and macronutrients analyzed included chlorophylls, carotenoids, magnesium (Mg), phosphorus (P), sulfur (S), potassium (K), and calcium (Ca). These leaf biochemical contents are important properties that reveal plant nutritional status and vegetation quality (Van Soest, 1994). The spectral analysis in this study focused on the wavelengths of 470–800 nm. This spectral region is of special interest in remote sensing of vegetation due to a significant absorption feature in the red spectral domain. A method of spectral standardization was developed to reduce the strong background effects in the leaf reflectance measurements for grassland plants. Chlorophyll and carotenoid concentrations were retrieved by inverting the physical model PROSPECT 5. The macronutrients were estimated empirically from specimens collected in the field, because foliar nutrients are not parameters of the PROSPECT 5 model, and cannot be retrieved through inversion of the physical model. PLS regression was used to build the linkages between the high-dimensional spectral predictor variables and the foliar biochemical contents.

## Materials and Methods

### Study Site

**Figure 1:**
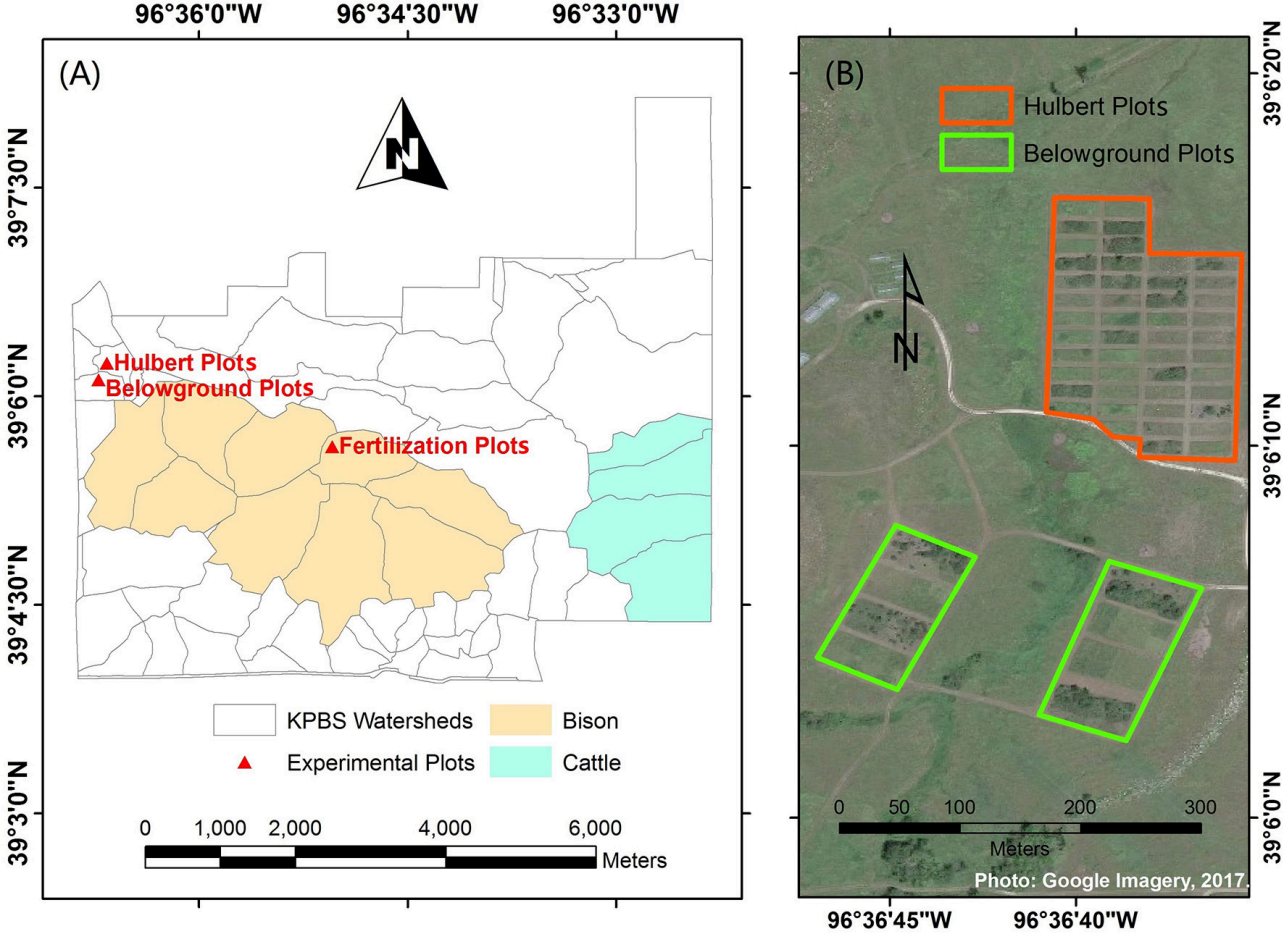
Study site at Konza Prairie Biological Station (KPBS). KPBS includes **(A)** more than fifty watersheds and **(B)** a variety of experimental plots, such as the Hulbert plots and Belowground plots.

This study was conducted at Konza Prairie Biological Station (KPBS, Figure 1), a tallgrass prairie site near Manhattan, Kansas, USA (39°05′N, 96°35′W). The vegetation at the site consists of more than 80% of grasses and a minor proportion of forbs. Dominant grass species include *Andropogon gerardii, Sorghastrum nutans, Panicum virgatum*, and *Schizachyrium scoparium*; forbs include *Aster ericoides, Psoralea tenuiflora, Solidago missouriensis, Soldiago rigida, Liaris aspera, Vernonia baldwinii*, and *Ambrosia psilostachya* (Collins and Calabrese, 2012).

KPBS is divided into more than fifty watersheds, in which varying combinations of fire and ungulate grazing treatments are replicated at the watershed level for long term investigations into the interactive processes among fire, large grazers, and vegetation communities. In addition, a variety of experiment plots are operated with differing fire or nutrition treatments for multiple research purposes. The foliar samples were collected from three of these experiment plots, including the Hulbert plots, the Belowground plots and the fertilization plots.

The Hulbert plots are managed to demonstrate the effects of fire on plant growth and species diversity. Each Hulbert plot measures 10 m × 25 m with a 5 m buffer, which is subjected to fire disturbances at an interval of 1, 2, 4, or 20 years. The Belowground plots are set up to investigate how varying combinations of fire, mowing, and fertilization affect both the above and below ground accumulation of biomass. There are two fire treatments (burned in spring and unburned), two mowing treatments (mowed and not mowed), and four fertilization treatments (additions of N, P, both N and P, and no fertilization addition) applied in a three-way factorial arrangement. Four replicates for each of the 16 treatment combinations are operated in a total of 64 plots. Each Belowground plot measures 12 m × 12 m (Callaham et al., 2002). The fertilization plots were developed at a bison (*Bison bison*) grazed site burned every 4 years, watershed N4B, in 2014. The plots were arrayed in four lines, two controlled (without applications of nitrogen fertilization) and two fertilized, which were alternately parallel arranged. Each line included five 2 m × 2 m plots with a one meter buffer. In each fertilized line, 0, 12, 24, 48, and 96 grams of ammonium nitrate (NH_3_NO_3_) were applied to each of the five plots, respectively, at the beginning of the growing season.

The treatments of fire and mowing have an immediate effect on the canopy structure. The fertilization additions affect the soil nutrient availability. All these treatments can influence the species composition in the canopy. The selection of these experiment plots allows a wide range of foliar biochemical contents to be sampled. The robustness of the modeling methods developed in this study can be examined across multiple plant functional groups.

### Data Collection

Field data were collected multiple times across seasons during the years of 2014–2016 (Table 1). In 2014–2015, the grasses and forbs were collected separately from the fertilization plots and the Hulbert plots; in 2016, mixed grassland plant types were collected from the Belowground plots. The datasets embodied variations from time, site, plant functional groups and measurement conditions, making it possible to evaluate the general robustness of the methodology in data analysis.

**Table 1:** Leaf sample datasets.

**Site**	**Fertilization plots**	**Hulbert plots**	**Belowground plots**
Date	July–September, 2014	June–September, 2015	July–September, 2016
Plant types (Sample size)	Forbs (20) Grasses (20)	Forbs (32) Grasses (32)	Mixed plant types (68)
**MEASUREMENT**			
Reflectance	ASD FieldSpec	ASD FieldSpec	ASD FieldSpec
**PIGMENTS**			
*Solvent*	Acetone 80%	DMSO	–
*Instrument*	Spectronic 20 Genesys	Spectronic 20 Genesys	–
Nutritional elements	–	X-Ray Fluorescence	X-Ray Fluorescence

–*, Not available*.

For each sample, around 5 grams of fresh leaves were randomly clipped from the canopy with a pair of scissors, and frozen in a cooler. Then the fresh leaf sample was divided into subsamples for measurements of reflectance, leaf pigments and nutritional elements in the laboratory. Hyperspectral reflectance data ranging between 350 and 2500 nm were measured using a leaf clip probe on an Analytical Spectral Devices (ASD) FieldSpec Pro portable Spectroradiometer (Analytical Spectral Devices, Boulder, CO, USA). During the leaf reflectance measurements, the ASD spectroradiometer was calibrated every half an hour. To determine chlorophyll and carotenoid concentrations, for each sample, a piece of leaf segment with an area of 0.559 cm^2^ was extracted from the leaf sample using a puncher, and then dipped into 10 ml 80% acetone or Dimethyl-Sulfoxide (DMSO) for 72 h dark storage (Gao, 2006). As the pigments were completely extracted, 3 ml solvent with the pigment extracts was transferred to a transparent cuvette and measured by a Spectronic 20 Genesys Spectroradiometer (Spectronic Instruments Inc., Rochester, NY, USA). The concentrations of chlorophyll a, b, and carotenoids in μg/ml were calculated using the empirical equations reported by Wellburn (1994), and scaled in μg/cm^2^ with the specified leaf sample area. The subsamples for analysis of macronutrients were dried in an oven for 72 h at 75°c, and then ground using a mortar and pestle. The resulting dry foliar powders were analyzed for element concentrations using a Bruker Tracer III-SD X-ray fluorescence Spectroradiometer (Bruker, Kennewick, WA, USA). Each sample of the dry foliar powders was measured three times, of which the average was used to reduce measurement errors. The X-ray fluorescence method for quantification of leaf nutritional elements is relatively new in plant analysis (Stephens and Calder, 2004; Towett et al., 2016). In our study, the leaf nutritional elements analyzed included Mg, P, S, K, and Ca. These elements are important plant nutrients. Their calibrations using the method of X-ray fluorescence measurement have been developed and proven reliable in previous studies (Towett et al., 2016).

### Spectral Standardization

**Figure 2:**
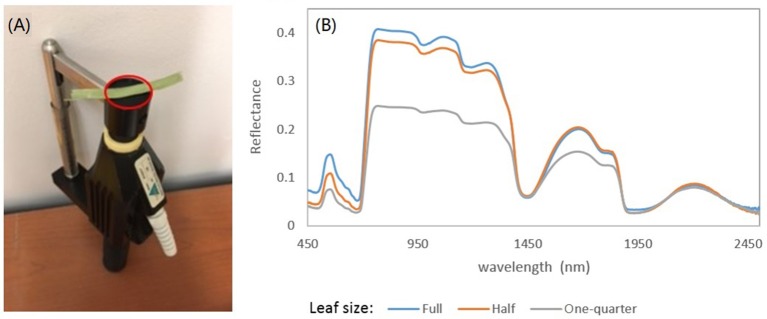
**(A)** ASD's leaf clip probe. Note that a narrow grassland leaf cannot cover the whole probe face. **(B)** The effects of leaf size on the measured reflectance spectra. The spectral signals can be shifted and stretched due to the background effects as the leaf cannot cover the whole probe face.

Spectral analysis in this study focused on the wavelengths of 470–800 nm. This spectral region includes a significant absorption feature in the red spectral domain, which is associated with photosynthetic pigments. In measurements of leaf reflectance for grassland plants, the background effects can be significant, given that the narrow leaves may not cover the whole leaf clip probe face of the ASD Spectroradiometer (Figure 2A). This irregular measurement may lead to a shift and stretch in the resulting spectrum (Figure 2B).

**Figure 3:**
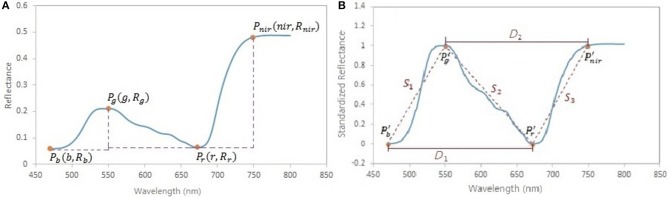
**(A)** Feature points in spectral standardization. *P*_*b*_ is the minimum point in the region of 470–520 nm; *P*_*g*_ is the maximum point in the region of 520–600 nm; *P*_*r*_ is the minimum point in the region of 600–720 nm; *P*_*nir*_ is the turning point in the region of 740–800 nm, where the first derivative is equal to 0. **(B)** Spectral slopes *S*_1_, *S*_2_, *S*_3_, and distances *D*_1_, *D*_2_ as variables potentially related to foliar biochemical contents. *P_b_*′, *P_g_*′, *P_r_*′, and *P_nir_*′ are the points on the scaled reflectance curve corresponding to the points *P*_*b*_, *P*_*g*_, *P*_*r*_, and *P*_*nir*_ on the original reflectance curve.

**Figure 4:**
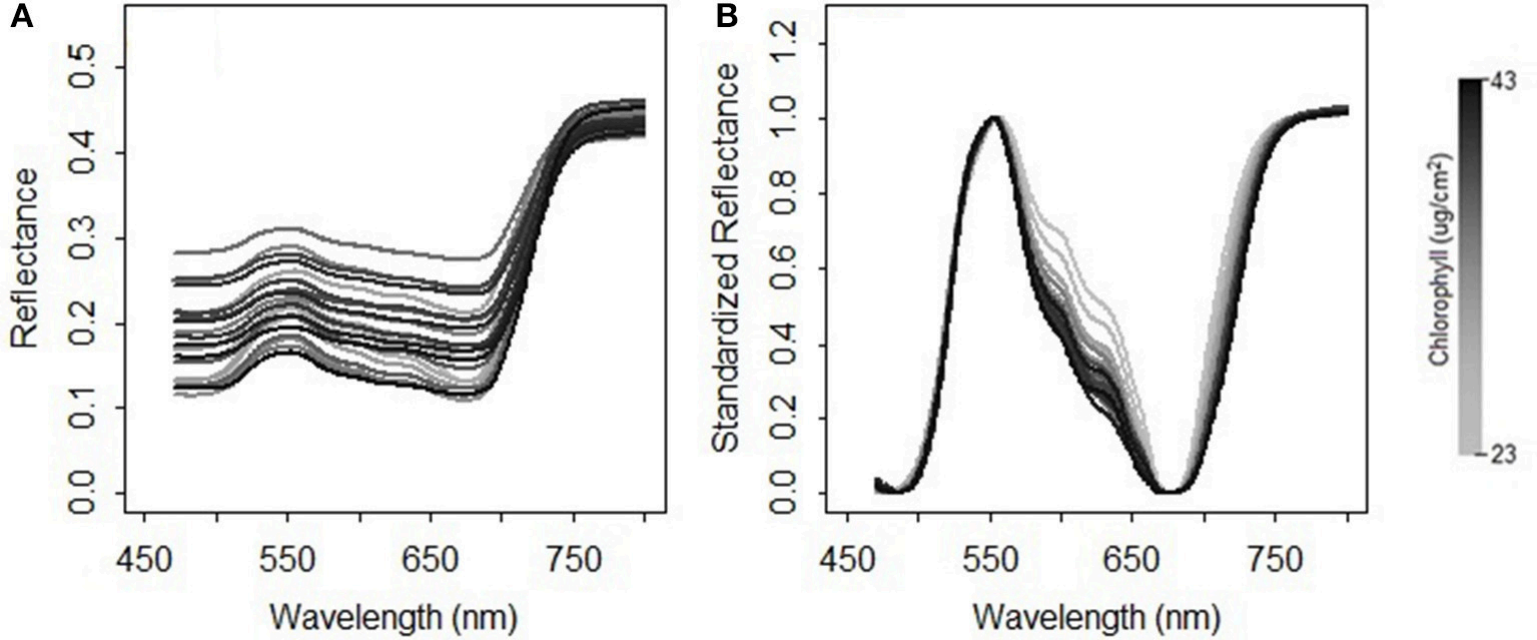
Comparison between **(A)** the original spectral measurements and **(B)** the standardized reflectance spectra for the grasses collected in 2015.

A spectral standardization method is developed to reduce the background effects in the leaf reflectance measurements. Four feature points are located on the original reflectance spectrum, including the local minima in the blue and red regions, the local maximum in the green region and the turning point in the near infrared region (Figure 3A). Based on these points, the original spectrum is scaled using a form of the normalized difference:

$$NDR_{i}=\{\begin{array}{c} \;\frac{R_{i}-R_{b}}{R_{g}-R_{b}},\;470\leq i<g\\ \frac{R_{i}-R_{r}}{R_{g}-R_{r}},\;g\leq i<r\\ \;\frac{R_{i}-R_{r}}{R_{nir}-R_{r}},\;r\leq i<800 \end{array}$$

where *NDR*_*i*_ is the scaled reflectance with a form of the normalized difference at the wavelength *i*; *b* is the wavelength of the minimal reflectance in the region of 470–520 nm; *g* is the wavelength of the maximum reflectance in the region of 520–600 nm; *r* is the wavelength of the minimum reflectance in the region of 600–720 nm; *nir* is the wavelength of the turning point in the region of 740–800 nm at which the first derivative is equal to 0; *R*_*i*_ is the reflectance value at the wavelength *i* nm. A comparison between the original reflectance and the scaled reflectance (Figure 4) shows that the spectral response pattern to the variation in the chlorophyll concentration is more evident in the scaled reflectance than that in the original spectra. This suggests that the spectral standardization method is feasible and practical.

In addition to the standardized reflectance by the normalized difference, spectral features that characterize the shape of the spectral curve, such as the slope (Lugassi et al., 2015), the red edge (Filella and Penuelas, 1994; Munden et al., 1994; Schut and Ketelaars, 2003; Mutanga and Skidmore, 2007), and the triangle surrounding the red absorption trough (Hunt et al., 2013), are considered important indicators of foliar biochemical contents. In this study, the absolute values of slopes across the wavelengths of *b*–*g, g*–*r, r*–*nir*, and distances across *b*–*r, g*–*nir* on the scaled reflectance spectral curve (Figure 3B) were included in spectral analysis:

$$\begin{array}{l} S_{1}=\frac{1}{g\;-\;b}\\ S_{2}=\frac{1}{r\;-\;g}\\ S_{3}=\frac{1}{nir\;-\;r}\\ D_{1}=r\;-\;b\\ D_{2}=nir\;-\;g \end{array}$$

where *S*_1_, *S*_2_, and *S*_3_ are the spectral slopes; *D*_1_ and *D*_2_ are the spectral distance variables. On the scaled reflectance spectral curve, the values at the wavelengths of *g* and *nir* are 1; the values at the wavelengths of *b* and *r* are 0.

### Retrieval of Leaf Pigments From PROSPECT 5

**Figure 5:**
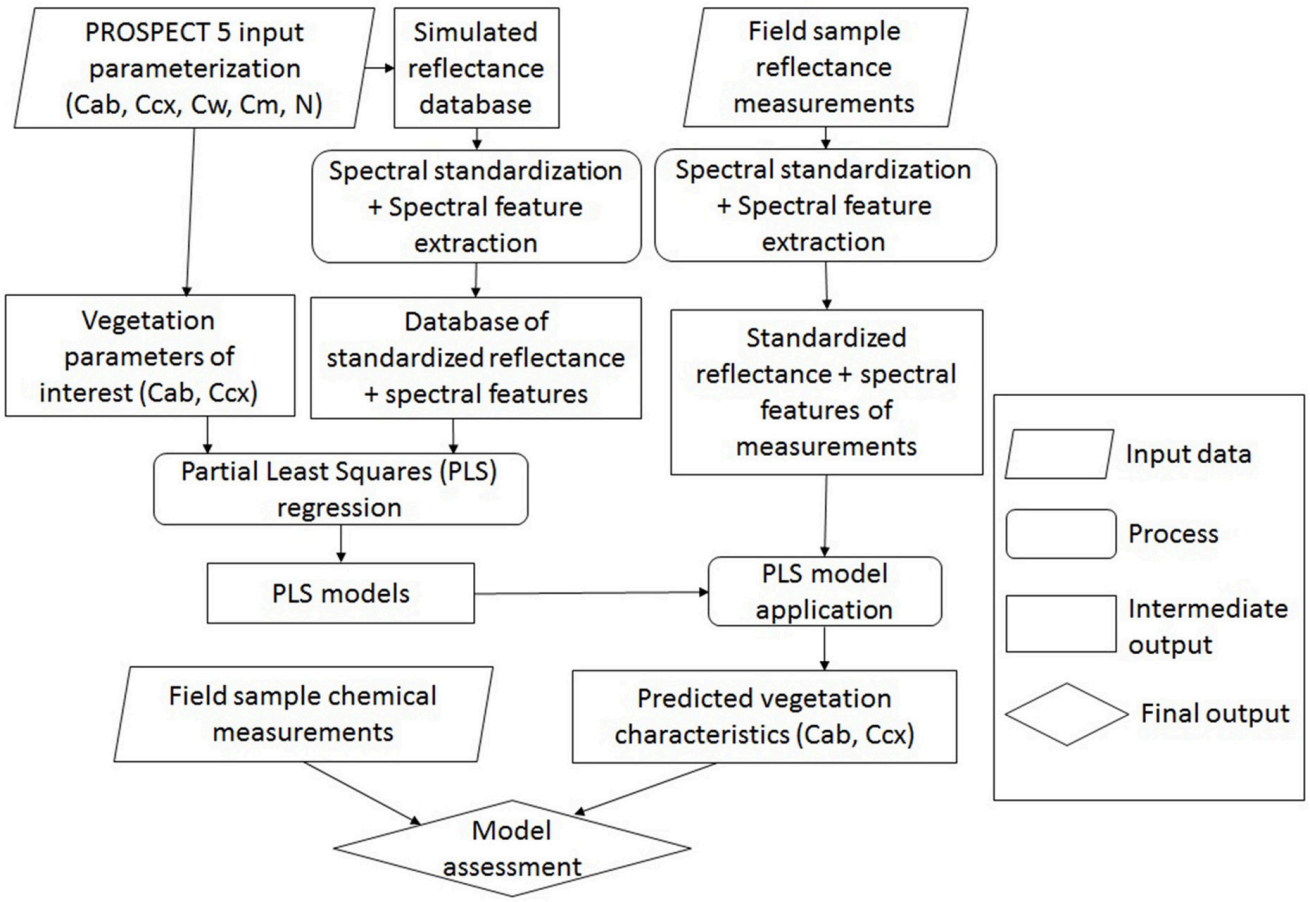
Overview of leaf pigment retrieval by inverting PROSPECT 5. The input parameters in PROSPECT 5 include chlorophylls (Cab), carotenoids (Ccx), water thickness (Cw), dry matter (Cm), and the leaf structure parameter (N). Concentrations of chlorophylls and carotenoids are of interest to be modeled.

Chlorophyll and carotenoid concentrations were retrieved by inverting the leaf radiative model PROSPECT 5 (Figure 5). A reflectance spectral database was simulated by varying the input parameters (Table 2), including chlorophylls (Cab), carotenoids (Ccx), water thickness (Cw), dry matter (Cm), and the leaf structure parameter (N). The output reflectance values at the wavelengths of 470–800 nm were standardized using the form of normalized difference, from which the spectral slope and distance features were extracted (see section Spectral Standardization). The resulting spectral variables, including *NDR*_470_–*NDR*_800_, *S*_1_–*S*_3_, *D*_1_ and *D*_2_ were related to chlorophyll and carotenoid concentrations in the original model parameterization through PLS regression. The PLS models were then applied to the standardized spectral variables of the field measurements for leaf pigment estimations. The predicted chlorophyll and carotenoid concentrations from the PLS models were compared with the laboratory chemical measurements for an assessment of the model performance. Model prediction accuracy was assessed by the root mean square error of prediction (RMSEP), the coefficient of variability (CV), and the index of agreement (*d*). RMSEP incorporates the bias (BIAS) and the standard error corrected from the bias (SEPC); CV is a measure of variation in relation to the mean, which indicates the magnitude of the error (Feret et al., 2008); *d* is a standardized measure of the degree of model prediction errors (Willmott, 1981):

$$\begin{array}{lll} RMSEP & = & \sqrt{\frac{\sum_{i=1}^{n}{\left(y_{i}^{{}^{\prime }}\;-\;y_{i}\right)}^{2}}{n}}\\ BIAS & = & \frac{\sum_{i=1}^{n}\left(y_{i}^{{}^{\prime }}\;-\;y_{i}\right)}{n}\\ SEPC & = & \sqrt{\frac{\sum_{i=1}^{n}{\left(y_{i}^{{}^{\prime }}\;-\;y_{i}\;-\;BIAS\right)}^{2}}{n}}\\ RMSEP^{2} & = & SEPC^{2}+BIAS^{2}\\ CV & = & 100\times \frac{SEPC}{\bar{y_{i}}}\\ d & = & 1\;-\;\frac{\sum_{i=1}^{n}{\left(y_{i}^{{}^{\prime }}\;-\;y_{i}\right)}^{2}}{\sum_{i=1}^{n}{\left(\left|y_{i}^{{}^{\prime }}\;-\;\bar{y_{i}}|+|y_{i}\;-\;\bar{y_{i}}\right|\right)}^{2}} \end{array}$$

where *y*_*i*_ is the measured value; y_*i*_′ is the predicted value; $\bar{y_{i}}$ is the mean of the measured values; *n* is the sample size. *d* varies between 0 and 1; a value of 0 indicates no agreement, and 1 indicates a perfect match.

**Table 2:** Input parameters and output in PROSPECT 5.

**Parameter**	**Range**	**Increment**
**INPUT**		
Chlorophyll (Cab)	6–60 μg/cm^2^	2 μg/cm^2^
Carotenoids (Ccx)	2–16 μg/cm^2^	2 μg/cm^2^
Water thickness (Cw)	0.008–0.02 g/cm^2^	0.004 g/cm^2^
Dry matter (Cm)	0.005–0.02 g/cm^2^	0.005 g/cm^2^
Leaf structure parameter (N)	1.5–3	0.5
**OUTPUT**		
Reflectance	470–800 nm	1 nm

### Empirical Estimation of Leaf Macronutrient

The foliar nutritional elements were modeled statistically from the standardized reflectance measurements using PLS regression. This procedure was not based on the PROSPECT model given that the foliar nutritional elements have not been calibrated as parameters in the radiative transfer process which the physical model describes. Half of the samples were used for model development, while the rest of the samples were used for model assessment. Both the model development and assessment datasets were required to cover the full range of the sampled nutritional elements.

## Results and Discussion

### Leaf Pigment Retrieval

#### Laboratory Chemical Analysis

Descriptive statistics for the leaf pigment measurements (Table 3) showed that chlorophylls ranged from 6.62 to 44.37 μg/cm^2^, and carotenoids ranged from 2.97 to 10.28 μg/cm^2^ across all the samples. These values were in a reasonable range, compared to those reported by Combal et al. (2003), le Maire et al. (2004), and Feret et al. (2008). Datasets collected from different plots and functional groups were slightly different in their statistical characteristics. The model robustness was allowed to be examined across different leaves with a wide range of leaf pigments.

**Table 3:** Descriptive statistics for the measured chlorophyll and carotenoid concentrations by laboratory chemical analysis.

	**Fertilization plot**		**Hulbert plot**	
	**Forbs**	**Grasses**	**Forbs**	**Grasses**
Sample size	20	20	32	32
**CHLOROPHYLLS (μg/cm**^**2**^**)**				
Min	28.37	27.04	6.62	24.92
Max	39.59	38.24	43.37	44.37
Mean	31.89	32.06	33.03	35.55
**CAROTENOIDS (μg/cm**^**2**^**)**				
Min	8.20	8.602	2.97	7.91
Max	10.28	10.12	8.97	10.12
Mean	9.08	9.149	7.65	8.90

#### Adjustment of the Leaf Structure Parameter in PROSPECT 5

**Figure 6:**
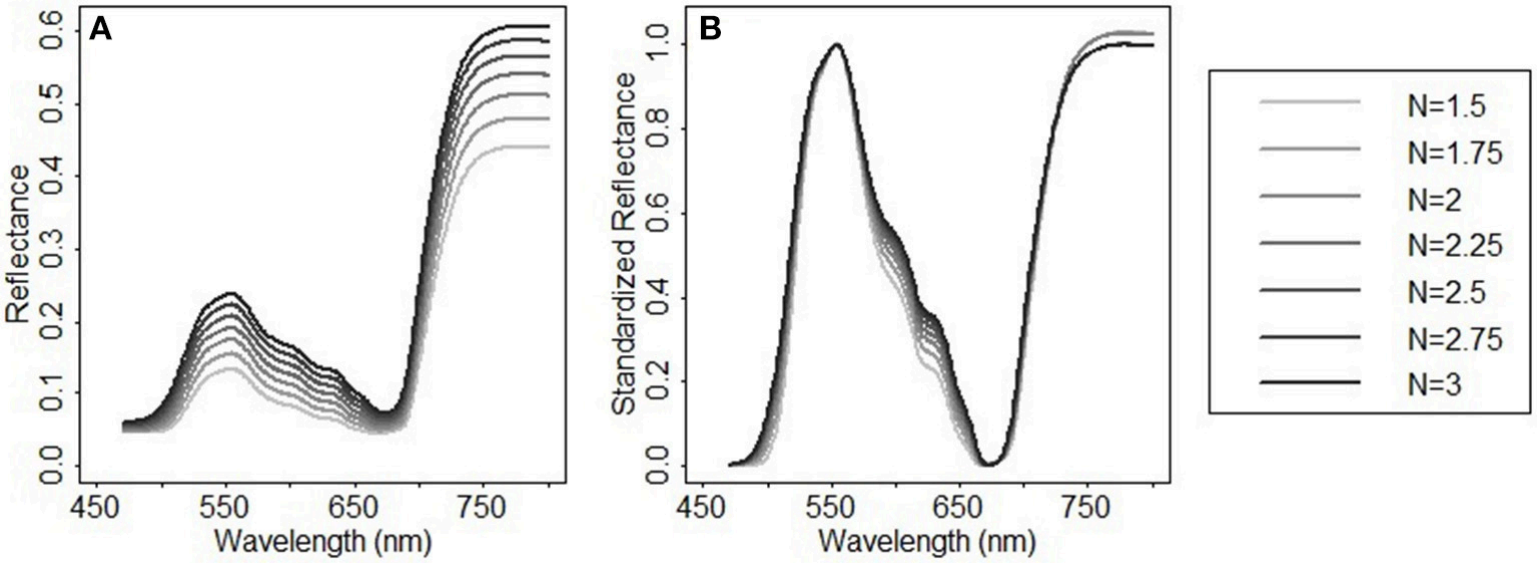
Spectral response patterns varying with the leaf structure parameter N in **(A)** the reflectance spectra simulated from PROSPECT 5 and **(B)** their corresponding standardized reflectance spectra. In the reflectance spectral simulation, Cab = 33 μg/cm^2^, Ccx = 9 μg/cm^2^, Cw = 0.014 g/cm^2^, Cm = 0.012 g/cm^2^, and N varies between 1.5 and 3 with a step of 0.25.

**Figure 7:**
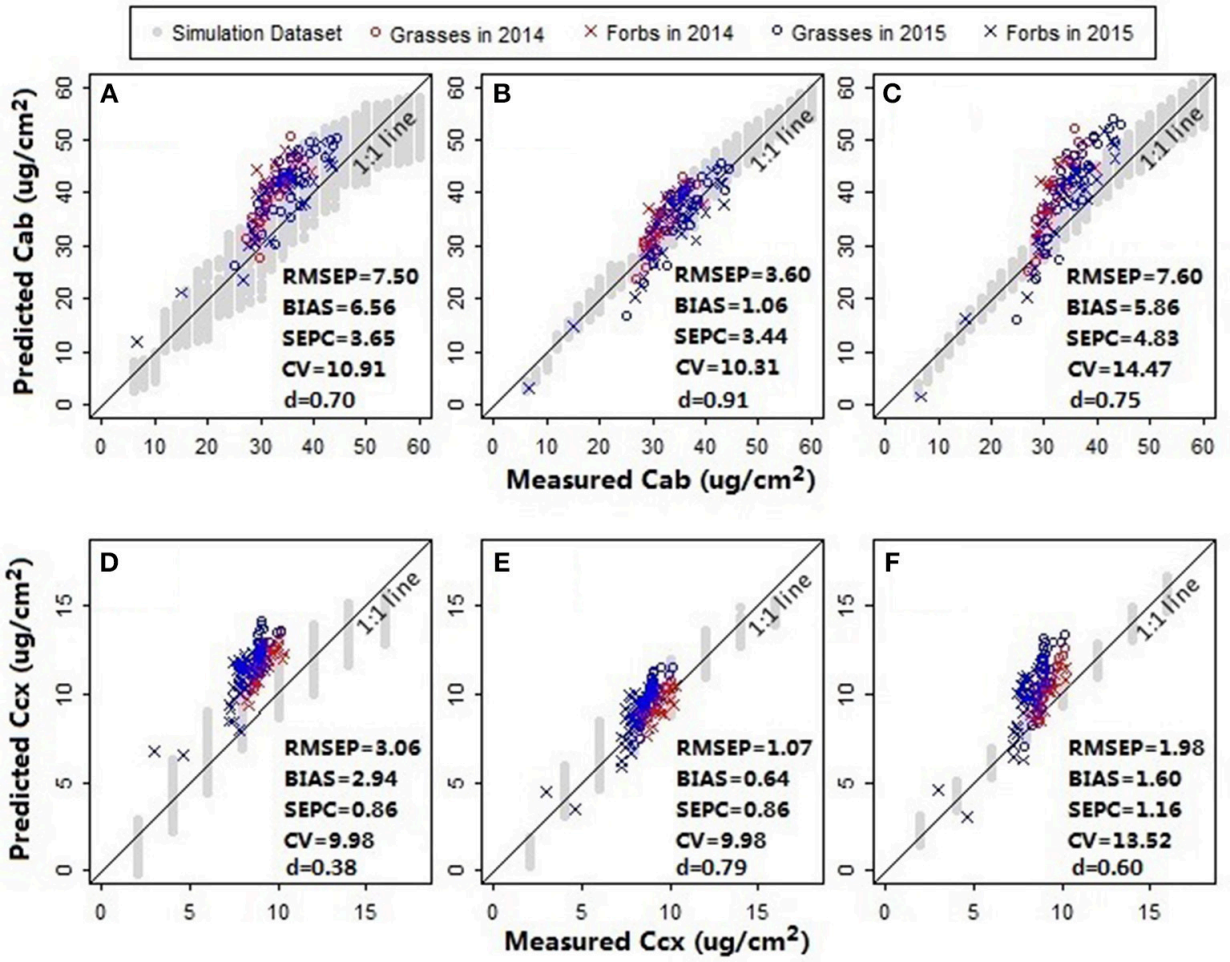
Model assessment for **(A–C)** chlorophylls and **(D–F)** carotenoids. Prediction accuracies of models with different leaf structure parameter ranges and spectral variables were compared. For the models in plots **(A,D)**, the leaf structure parameter N ranged between 1.5 and 3; the spectral variables *NDR*_470_–*NDR*_800_, *S*_1_–*S*_3_, *D*_1_, and *D*_2_ were included as the manifest explanatory variables for PLS regressions. In plots **(B,E)** N was adjusted within a range between 1.7 and 1.9; the spectral variables were the same with that in plots **(A,D)**. In plots **(C,F)** N ranged between 1.7 and 1.9; the manifest explanatory variables included *NDR*_470_–*NDR*_800_, whereas the slope and distance spectral variables were excluded. The RMSEP, BIAS, SEPC, CV, and *d* were calculated for the pooled samples collected from the fertilization plots in 2014 and the Hulbert plots in 2015. All the models were built using the first three PLS factors.

In addition to chlorophylls and carotenoids, the leaf structure parameter has a significant effect on the spectral shape in the visible and near infrared region (le Maire et al., 2004). A systematic change in the spectral response patterns due to variations in the leaf structure parameter can be seen both in the original reflectance spectra simulated from PROSPECT 5 and their corresponding standardized reflectance spectra (Figure 6). In the original parameterization, the leaf structure parameter N ranged between 1.5 and 3. The resulting predictions of chlorophylls and carotenoids were generally overestimated with the biases of 6.56 μg/cm^2^ (Figure 7A) and 2.94 μg/cm^2^ (Figure 7D), respectively. As N was adjusted within 1.7–1.9, the model biases were reduced, and the model prediction accuracy and the agreement statistics improved substantially (Figures 7B,E). This result indicates that a proper selection of the N range is essential for accurate retrieval of leaf biochemical contents using the PROSPECT model.

#### Spectral Feature Selection by PLS Regression

For the leaf pigment retrieval models in this study, the first three PLS factors were adequate to account for much of the variance in the data and led to relatively high prediction accuracy. The available predictors included the standardized continuous reflectance variables *NDR*_470_–*NDR*_800_, the spectral slopes *S*_1_–*S*_3_, and the distance variables *D*_1_ and *D*_2_. These predictor variables were different in characteristics, forms and magnitudes. Their importance to the corresponding PLS model is of interest.

**Figure 8:**
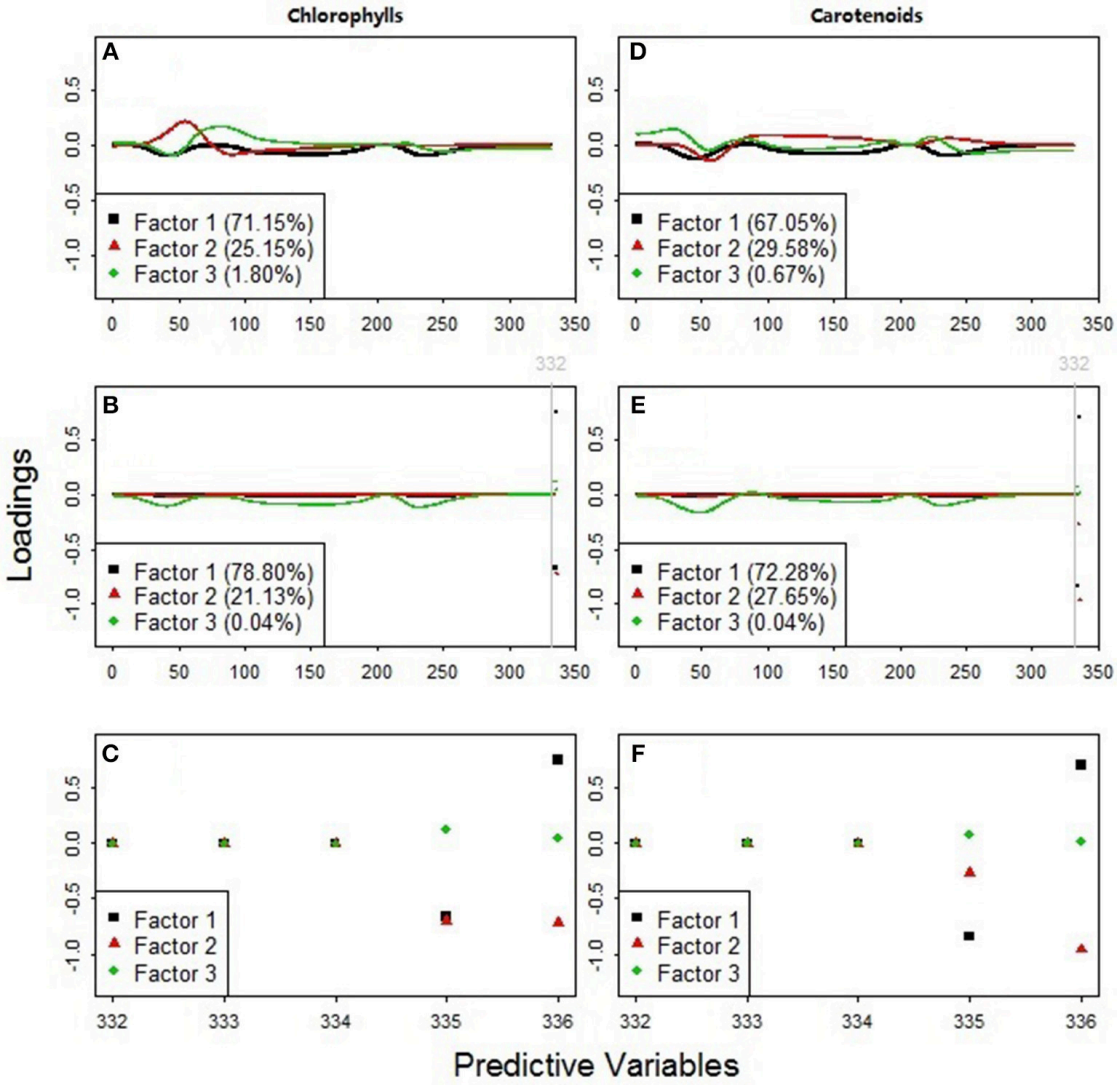
Predictor variable loadings for the PLS factors used to estimate **(A–C)** chlorophylls and **(D–F)** carotenoids. The models in plots **(A,D)** included 331 standardized continuous reflectance variables, *NDR*_470_*-NDR*_800_, as the predictors. The models in plots **(B,E)** included all the available predictors, *NDR*_470_*-NDR*_800_, *S*_1_*-S*_3_, *D*_1_, and *D*_2_. Plots **(C,F)** zoomed in on the loading distributions among the predictors 332–336 (the slope and distance predictor variables, *S*_1_*-S*_3_, *D*_1_, and *D*_2_).

Results showed that the models including all the available predictors (*NDR*_470_–*NDR*_800_, *S*_1_–*S*_3_, *D*_1_ and *D*_2_, see Figures 7B,E) had higher prediction accuracy and agreement statistics than those including only the standardized continuous reflectance variables (*NDR*_470_–*NDR*_800_, see Figures 7C,F). With the slope and distance predictor variables included, high loadings occurred at the distance variables in the first two PLS factors, which accounted for more than 99% variance in the data (Figures 8B,C,E,F). This indicates a significant effect from the distance spectral variables (*D*_1_ and *D*_2_) on predicting leaf pigments. The distance variables are comparable with the leaf pigment spectral features, such as the red edge (Filella and Penuelas, 1994; Munden et al., 1994; Schut and Ketelaars, 2003; Mutanga and Skidmore, 2007) and the red absorption triangle (Hunt et al., 2013), which are based on the positions of specific spectral feature points. The magnitude of the distance variables is far higher than that of the standardized continuous reflectance variables. This may be a factor that results in greater loadings at the distance spectral variables. Although the distance spectral variables are different from the standardized continuous reflectance variables in characteristics, forms and magnitudes, the addition of the distance variables in this way as the predictors substantially improved the model accuracy and robustness.

The PLS loading distributions among the standardized continuous reflectance variables revealed useful hyperspectral features for detecting grassland plant quality. The loadings of the first PLS factors in the models with only the standardized continuous reflectance predictors (black squares in Figures 8A,D) are similar to that of the third PLS factors in the models with all the available predictors (green diamonds in Figures 8B,E). This result suggests that the feature selection and integration among the standardized continuous reflectance predictor variables via such a loading pattern can be an important indicator of leaf chlorophyll and carotenoid concentrations.

### Leaf Macronutrient Estimation

#### Laboratory X-ray Fluorescence Analysis

The leaf macronutrient concentrations were measured by an X-ray fluorescence spectroscopy. The elements analyzed included Mg, P, S, K, and Ca. These nutritional elements are integral constituents of plant biomass and relevant for grazer nutrition. The samples were divided almost equally for modeling and validation. The descriptive statistics (Table 4) showed that the range and mean of the modeling dataset were consistent with that of the validation dataset, suggesting a proper selection of the empirical modeling and validation datasets.

**Table 4:** Descriptive statistics of the foliar nutritional element concentrations for the modeling and validation datasets.

**Element**	**Modeling**				**Validation**			
	**Sample size**	**Min**	**Max**	**Mean**	**Sample size**	**Min**	**Max**	**Mean**
Mg	62	0.119	0.257	0.173	56	0.122	0.262	0.177
P	65	0.033	0.172	0.091	61	0.047	0.169	0.094
S	64	0.040	0.154	0.087	56	0.045	0.144	0.087
K	65	0.363	2.256	1.102	56	0.377	2.324	1.115
Ca	60	0.255	1.966	0.790	56	0.281	1.847	0.788

*The number of the samples used in modeling and validation was slightly less than the foliar sample size in the field data collection due to the loss in the laboratory measurements and the outliers in the spectral modeling process*.

#### Empirical Modeling by PLS Regression

**Figure 9:**
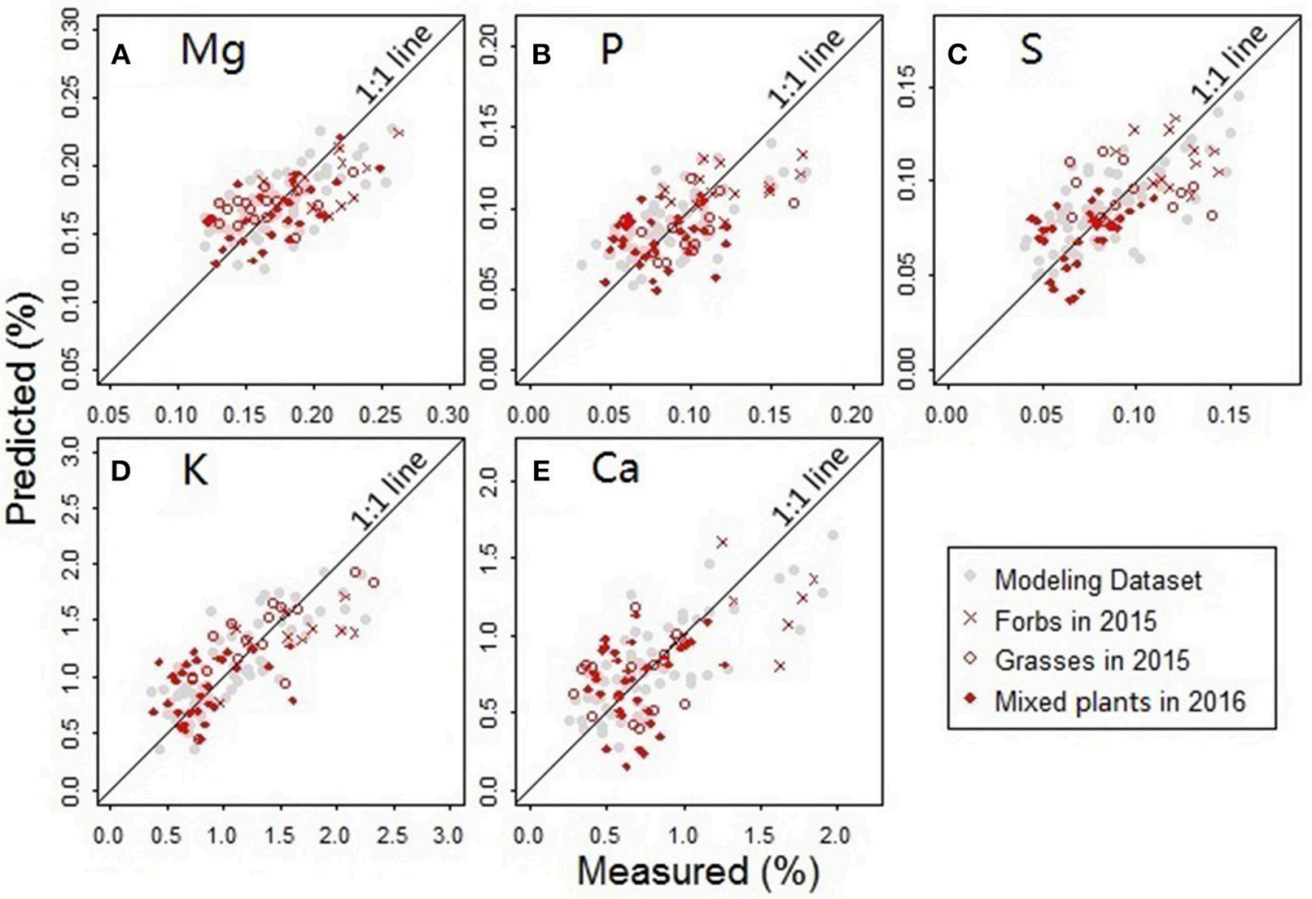
Comparisons between the measured and predicted nutrient concentrations for the elements **(A)** Mg, **(B)** P, **(C)** S, **(D)** K, and **(E)** Ca. Samples used in the analysis included the forbs and grasses collected from the Hulbert plots in 2015 and the mixed plant types collected from the Belowground plots in 2016.

The predictors for PLS regression modeling of the plant nutrients included *NDR*_470_–*NDR*_800_, *S*_1_–*S*_3_, *D*_1_ and *D*_2_. In the best-performing models (Figure 9), there are no evident patterns observed among the multiple plant functional groups, which indicates that the models are robust across different plant forms. In the assessment of the model performance (Table 5), the RMSEP values for model-development are similar to that for model-validation; the bias values in the validation procedure are at low levels. This consistency between the modeling and validation procedures verifies the model prediction capability. The CV value is relatively low for the model of the element Mg, but high for the model of Ca, indicating the magnitude of the prediction error is low for Mg, but high for Ca. The *d* values are at a generally high level, indicating a good agreement between the predicted values and the measured values.

**Table 5:** Assessment of the PLS models for nutrient predictions.

**Element**	**Mg**	**P**	**S**	**K**	**Ca**
**MODELING**					
Number of factors	8	9	6	7	8
RMSEP	0.0246	0.0226	0.0189	0.2877	0.2555
**VALIDATION**					
RMSEP	0.0269	0.0249	0.0224	0.3282	0.3257
BIAS	0.0051	0.0040	0.0026	0.0044	0.0187
SEPC	0.0264	0.0246	0.0223	0.3282	0.3251
CV	14.9437	26.1488	25.5306	29.4423	41.2515
*d*	0.7352	0.6910	0.7597	0.8327	0.6865

The nutritional element models generally require six to nine PLS factors to achieve an acceptably low prediction error when there is no evident modeling bias observed. Compared to the three PLS factors in the leaf pigment retrieval models, an increased number of factors in the nutrient models make the nutrient predictions more complex. This finding implies that the spectral modeling of the nutrient concentrations depends more on the finely resolved hyperspectral features (Mutanga et al., 2004a).

#### Correlations Between Leaf Biochemical Constituents

Correlations (Pearson's r) between leaf biochemical constituents were calculated for the Hulbert plot dataset, in which both the leaf pigments and the nutritional elements were quantified through the laboratory analysis (Table 6). The strong correlations between chlorophylls and carotenoids are consistent with the observations in previous studies indicating that chlorophylls and carotenoids are co-varying in nature and statistically dependent (Feret et al., 2008). Most of the plant nutritional elements are significantly correlated. This association is understandable because the macronutrients are collectively responsible for plant metabolic processes (Mutanga et al., 2004b).

**Table 6:** Correlations between leaf biochemical constituents.

	**Cab**	**Ccx**	**Cab:Ccx**	**Mg**	**P**	**S**	**K**	**Ca**
Cab	1							
Ccx	0.80^*^	1						
Cab:Ccx	0.71^*^	0.18	1					
Mg	−0.10	−0.53^*^	0.23	1				
P	0.17	−0.19	0.35^*^	0.53^*^	1			
S	0.13	−0.23	0.32^*^	0.49^*^	0.60^*^	1		
K	0.32^*^	−0.12	0.50^*^	0.39^*^	0.63^*^	0.63^*^	1	
Ca	−0.12	−0.62^*^	0.27	0.79^*^	0.26	0.31^*^	0.20	1

* *Statistically significant at the 95% confidence level: p < 0.05*.

Relationships between leaf photosynthetic pigments and nutritional elements in this native grassland study system are of interest. Chlorophylls are positively correlated with the element K. Carotenoids are negatively correlated with Mg and Ca. There are no other statistically significant correlations between the leaf pigments and the nutritional elements. However, the ratio of chlorophylls to carotenoids shows positive correlations with the elements P, S, and K. This is consistent with previous studies which reveal that the ratio of chlorophylls to carotenoids can be an important index that reflects plant phenology and nutritional status in tightly-controlled agricultural systems (Feret et al., 2008; Yang et al., 2010). According to results of our study, the ratio of chlorophylls to carotenoids is also found useful for detecting general vegetation nutrition and forage quality across dominant grasses and forbs in a natural tallgrass prairie system.

### Forage Quality Across Plant Functional Groups

The hyperspectral analysis methods developed in this study were verified to be robust and reliable across different plant functional groups. There was no evident bias found among models for grasses, forbs, and mixed plant types in the retrieval of leaf pigments and macronutrients. This reveals a limitation in differentiation between grasses and forbs by using the spectral features analyzed in this study. However, classification of plant types and functional groups is necessary in determining grassland forage quality for Plains bison in tallgrass prairies. Bison typically select palatable grass species and avoid forbs (Plumb and Dodd, 1993; Raynor et al., 2016). Analysis of grassland nutrient distribution without consideration of plant types and functional groups may lead to incorrect interpretations of relationships between forage quality and bison grazing patterns. Further researches on discrimination between grasses and forbs through field measurements or texture analysis (Petrou et al., 2015) with remote sensing imagery are essential for fully understanding the interplay between vegetation resources and ungulate grazers.

## Conclusions

Results of this study show that the hyperspectral features in the spectral region of 470–800 nm are useful for detecting concentrations of leaf pigments and nutritional elements. A spectral standardization method using a form of normalized difference is developed and proved effective to reduce the significant background impact in measurements of leaf reflectance for grassland plants. In this method, four feature points are highlighted, including the nadirs in the blue and red regions, the green peak and the turning point in the near infrared region. The positions and reflectance values of these feature points provide useful information for estimating leaf pigments.

In retrieval of leaf pigments from PROSPECT 5, the leaf structure parameter has a significant effect on the spectral response pattern. A proper selection of the range of the leaf structure parameter can reduce much of the bias in model validation and improve model prediction accuracy. This study documents that a range of leaf structure parameter from 1.7 to 1.9 is reasonable for common forbs and grasses in tallgrass prairies. In inversion of PROSPECT 5, PLS regression shows the capability of building the linkages between the high dimensional spectral variables and the vegetation parameters. The advantage of using PLS regression is that the spectral features relevant to the vegetation parameters of interest can be selected and integrated effectively from a wide range of available spectral predictor variables.

Development of PLS regression models for the leaf nutrients demonstrates that a reasonable selection of the modeling and validation datasets is critical to improving prediction accuracy of the empirical models. The nutrient models require more PLS factors to achieve an acceptable level of model accuracy than the models developed for retrieval of leaf pigments. This finding implies that spectral modeling of the nutrients is more complex and depends more on the finely resolved spectral features.

Promising methods to quantify leaf pigments and nutritional elements using the hyperspectral analysis were developed in this study. The model prediction accuracy is comparable with those reported by Feret et al. (2008) for leaf pigment retrieval and Mutanga et al. (2004b) for nutritional element estimation. Further, this study examined relationships between leaf photosynthetic pigments and nutritional elements, providing a comprehensive assessment of leaf nutrition status for grassland forbs and grasses. It is found that the leaf photosynthetic pigments are significantly correlated with part of the nutritional elements. The ratio of chlorophylls to carotenoids is informative to reflect the plant phenology and nutrition status (Feret et al., 2008; Yang et al., 2010). These findings provide insight into the use of pigment-related vegetation indices as indicators of vegetation quality. The spectral models developed in this study are robust across different plant types and measurement conditions. These results at the leaf level are of great value as a preliminary step to mapping the forage quality in grassland canopies from reflectance data collected by airborne or satellite sensors.

## Author Contributions

This research is part of BL doctoral dissertation. BL collected the field data, conducted the data analysis, and wrote the majority of the manuscript. DG supervised the research and helped with field data collection and laboratory chemical analysis. ER and AJ contributed to field experiment design. AJ was the Principal Investigator of the funded project of which this research was a part, and research grant provided by Guangdong University of Technology.

### Conflict of Interest Statement

The authors declare that the research was conducted in the absence of any commercial or financial relationships that could be construed as a potential conflict of interest.
